# Chat generative pre-trained transformers era: pros and cons between nursing researchers in Egypt

**DOI:** 10.1186/s12912-025-03332-1

**Published:** 2025-06-24

**Authors:** Nafisa Moustafa Abdallah Elpasiony, Elsayed Mahmoud Sabek, Safaa Sayed Mustafa Ibrahim

**Affiliations:** 1 Medical Surgical Nursing, Faculty of nursing, Beni-Suef University, Beni Suef, Egypt; 2 Critical Care and Emergency Nursing, Faculty of Nursing, Beni-Suef University, Beni Suef, Egypt; 3 Faculty of Nursing, Al-Baha University, Al-Baha, Saudi Arabia; 4 Community Health Nursing, Faculty of Nursing, Beni-Suef University, Beni Suef, Egypt

**Keywords:** ChatGPT, Pros, Cons, Nursing researchers

## Abstract

**Background:**

An artificial intelligence chatbot called Chat Generative Pre-Trained Transformers (ChatGPT) was created by OpenAI. It gained a lot of interest and attention from the scientific and academic sectors since its November 2022 launch.

**Aim:**

To identify ChatGPT pros and cons between nursing researchers in Egypt.

**Methods:**

A descriptive (cross sectional) research design was conducted on a convenient sample of 1001 nursing researchers from faculties of nursing related to the Supreme Council of Universities. Two tools were used: demographic and technical characteristics, and nursing researchers’ opinion questionnaire.

**Results:**

The majority of the participants **(**81.4%) thought that using ChatGPT had significant advantages. However, 44.7% of the cons were disclosed. Almost two-thirds of nursing researchers stated that they are concerned about patient confidentiality (65.6%), that it could lead to incorrect conclusions (68.8%), and that it could have medicolegal repercussions (68.6%). As a result, they are reluctant to use AI chatbots in healthcare decisions (67.8%).

**Conclusion and recommendations:**

ChatGPT had benefits but at the same time associated with drawbacks and needs to be used wisely to avoid these drawbacks. Enhance ChatGPT’s ability to foster reflective practice to enhance decision-making and critical thinking while bridging the theoretical and practical knowledge gaps in nursing research.

**Supplementary Information:**

The online version contains supplementary material available at 10.1186/s12912-025-03332-1.

## Introduction

The evolution of artificial intelligence (AI)-based health care technology systems has been witnessed a surge during the last decade. An AI-based chatbot called chat Generative Pre-trained Transformer (ChatGPT) was launched in November 2022 and is currently being used in nursing practice, research, and education. ChatGPT developed by OpenAI and is based on GPT language model technology. It is an incredibly smart chatbot that can respond to basic inquiries and carry out more complex tasks as it can provide information, answer questions, admit its mistakes, decline improper requests language translation, text generation, text summarization and sentiment analysis [1].

Through leveraging of its vast data stores and effective architecture, ChatGPT is able to comprehend and analyze user queries before producing pertinent answers in language that is almost identical to that of a human. ChatGPT can enhance its performance over time using deep learning techniques. Apart from its practical uses, ChatGPT is a noteworthy advancement in the fields of natural language processing (NLP) and AI due to its capacity to produce language that is human-like and accomplish challenging tasks [2].

ChatGPT has drawn the attention of many users, including those in nursing field due to its popularity, accessibility, and diversity. Its launch marked a key milestone as a strong tool across multiple sectors. Its great use and broad adoption can be attributed to these features [3]. Digital technologies pledge to reduce nurses’ workloads and increase the quality of care. Legitimacy of this technology hinges on their influence on nurses’ work, safety, and patient outcomes [4].

Furthermore, ChatGPT has the ability to offer prompt, precise, and tailored answers to a variety of medical queries as it allows nurses to interact with patients and coworkers in real time while also finding information about drugs, procedures, and patient care fast. In addition, ChatGPT can help nurses stay up-to-date on the latest research and best practices in nursing. Additionally, ChatGPT can assist nurses in being up-to-date of the most recent nursing research and best practices. This can guarantee that nurses are giving the best care possible and assist them in making well-informed decisions about patient care [5].

While nursing students can use ChatGPT to formulate patient scenarios and to practice their skills in a safe and controlled environment. This can reduce the risk of errors, and increase students’ confidence [6]. Moreover, it is considered a powerful tool especially for researchers whose first language isn’t English when used appropriately, for instance, it can generate idea, suggest future research, consume time of extensive search for literature review, analyze data, rewrite scientific articles and, thus, researchers can deliver their thoughts in a better way [7].

Despite ChatGPT is advantageous in conversational and writing tasks, as well as the precision of the expected results, it is associated with drawbacks. It could be linked to the risks of plagiarism, misinterpretations of queries, and technical constraints. It might not possess the context of the suggested research. In addition, researchers may become over-reliant on it for creating scientific contents, potentially resulting in a lack of critical thinking and originality in scientific writing. Contents generated by ChatGPT might be irrelevant, inconsistent, lacking in coherence, and personal views. Furthermore, security concerns and the possibility of cyber-attacks arising from the dissemination of misinformation must also be taken into account [8, 9].

Following the broad launch of ChatGPT, debates of its utilization were aroused. This may be due to the innate resistance of the human mind to any change [10]. The debates enforced the researchers to conduct this study to identify pros and cons of using ChatGPT.

## Methods

### Aim of the study

To identify ChatGPT pros and cons between nursing researchers in Egypt.

#### Research questions

1. What are the nursing researchers’ opinions toward using ChatGPT in Egypt?
2. What are the barriers of applying artificial intelligence in health care researches?
3. Are there relations between participants’ demographic data and their opinion toward ChatGPT utilization?

### Research design, sample, and sampling

A descriptive (cross sectional) research design was conducted on a convenient sample of 1001 nursing researchers from faculties of nursing in 23 university, 16 governmental university and 7 private university related to the Supreme Council of Universities of Egypt with the following inclusion criteria:

All nursing researchers including those who are doing research for graduation project and accept to participate in the survey.

The faculties of nursing from all over Egypt. Sample size was estimated using epi info seven program. We selected a 99.9% confidence level for our sample size calculation to ensure maximum statistical rigor and reliability of the results. The higher confidence level minimizes the probability of Type 1 error which is especially important in health-related research where accurate estimation is vital for clinical or policy decisions. Furthermore, a more stringent confidence interval guarantees the study results’ increased reliability and defensibility, especially considering the possible significance of the findings [11].

Population size = 5000.

Expected frequency = 50%.

Acceptable margin of error = 5%.

Confidence coefficient = 99.9%.

The estimated sample size was calculated to be 890 nursing researchers, while 1001 participants completed the survey.

### Tools

The survey instruments used were an online questionnaire developed by the researchers to identify the pros and cons of using ChatGPT between nursing researchers in Egypt. An extensive review of related researchers was part of the development process. Microsoft Forms, a secure online platform, was used to distribute the questionnaire, guaranteeing participants’ ease and accessibility. Participants received the survey link over email and other secure ways. The questionnaire was composed of two instruments.

### Demographic and technical characteristics

First part including demographic characteristics that assessed researchers age, gender, educational degree, affiliation, and specialty. Second part including technical characteristics that assessed participants computer skills, knowledge about artificial intelligence, and experience with using ChatGPT.

### Nursing researchers’ opinion questionnaire

After reviewing related studies [12–15, 10, 16], the researchers developed this questionnaire to identify participants’ viewpoint for the pros, cons, and barriers of ChatGPT utilization. This questionnaire consists of two parts. The first part, 17 items for the pros and 16 items for the cons, each item answered with a three-point Likert scale; 0 = no, 1 = maybe, 2 = yes. The pros comprised items like efficiency of scientific writing; translation purposes, time and cost saving, accessibility and productivity. The highest score of pros is 34 which indicates positive view. While the cons included items such as insecurity, bias, content not original, ethical concerns, and plagiarism. The highest score is 32 which indicates negative view. The second part incorporates the frequency of 10 items related to the barriers of artificial intelligence utilization in health care researches. These items like unavailability, fear from wrong decisions, and resistance. The detailed items of the questionnaire in attached file https://forms.office.com/r/FVvp4V9WfE.

### Tools validity and reliability

Following the development of the questionnaire, three experts in medical surgical nursing, critical care nursing, and community health nursing assessed the questionnaire’s validity to make sure it was clear and pertinent. Furthermore, the internal consistency was evaluated using Cronbach’s alpha test α = 0.8293 which reflects high internal consistency of the questionnaire.

### Pilot study

Over the course of one month, it was carried out on 100 nursing researchers, 10% of the study’s total participants, to determine the instruments’ application and clearance. The study does not include the pilot study participants.

### Data collection procedures

Initially, a written approval was obtained from the ethics of research committee of the faculty of medicine, Beni-Suef University, Egypt (Ethics ref. FMBSUREC/03102023/Ibrahim) to conduct the study. Nursing researchers who partake in the survey, were recruited from faculties of nursing affiliated with Supreme Council of universities. Through email and other official WhatsApp channels of the faculties, each participant received an electronic self-administered questionnaire, an information sheet, and a consent form. The researchers explained the nature and purpose of the study, emphasized that confidentiality was guaranteed, and the involvement in the study or the decision to withdraw is completely voluntary. Moreover, participants were informed that the data will not be reused for any other research purpose without their permission. All these rights of participation were emphasized on the first page of the survey. Access and completion of the online survey were granted only after participants provided electronic informed consent through the questionnaire platform. Approximately 15–20 min are required to complete the questionnaire. The researchers were available for any inquiries from the study participants. A solitary reminder was dispatched through the same communication channels that used initially to enhance response rates and to avoid any feeling of coercion. Data collection was conducted from October 2023 to September 2024.

### Analysis of the data

Data were analyzed utilizing IBM SPSS software package version 20.0. (Armonk, NY: IBM Corp). Qualitative data were represented using number and percent. The Kolmogorov-Smirnov test was employed to verify the normality of distribution. Quantitative data were demonstrated using range (minimum and maximum), mean, standard deviation and median. The significance of the results obtained was evaluated at the 5% level. The student t-test was employed for comparing normally distributed quantitative variables between two groups, while the F-test (ANOVA) was used for comparisons among more than two groups of normally distributed quantitative variables. To determine internal consistency, Cronbach’s alpha test was applied.

Table 1. displays that 86.5% of nursing researchers were between 18 to less than 30 years old with 64.0% of them were female. Moreover, 56.0% were bachelor’s degree with 34.5% were from medical surgical nursing department.

**Table 1:** Percentage distribution of the participants according to demographic data (*n* = 1001)

Demographic data	No.	%
1. Age		
18 < 30 years	866	86.5
30 < 40 years	120	12.0
40 < 60 years	15	1.5
Min. – Max.	18.0–49.0	
Mean ± SD.	23.4 ± 5.4	
Median	20.0	
2. Gender		
Male	360	36.0
Female	641	64.0
3. Level of education		
Bachelor’s Degree	561	56.0
Master	275	27.5
Doctorate	122	12.2
Ass. Prof.	21	2.1
Professors	22	2.2
4. Specialty		
Medical Surgical nursing	344	34.4
Critical care nursing	204	20.4
Obstetric nursing	55	5.5
Pediatric nursing	106	10.6
Community health nursing	133	13.3
Administration nursing	108	10.8
Psychiatric nursing	51	5.1

SD: Standard deviation Ass. Prof.: Associate professor

Table 2. represents that 63.1% of the participants had good knowledge and expertise of computer. Furthermore, 64.3% working on current research less than one year. Besides, 51.2% had experience with ChatGPT and 51.8% used ChatGPT in the current research.

**Table 2:** Percentage distribution of the participants according to technical data (*n* = 1001)

Technical data	No.	%
1. Level of computer knowledge and expertise		
Good	632	63.1
Fair	247	24.7
Poor	122	12.2
2. How long have you been working on current research?		
Less than 1 years	644	64.3
1 < 2 years	200	20.0
2 < 5 years	79	7.9
5 < 10 years	33	3.3
More than 10 years	45	4.5
3. Do you have any experience with ChatGPT?		
Yes	513	51.2
No	488	48.8
4. Did you use ChatGPT in current research?		
Yes	519	51.8
No	482	48.2

A large percentage of nursing researchers exhibits positive opinion toward pros of using ChatGPT. 81.7% of them showed that ChatGPT helps to save time, 77.5% reported that ChatGPT can be a useful tool for researchers, and 76.8% said that ChatGPT helps to provide easily accessible and understandable information. While 59.9% stated that ChatGPT able to generate plagiarism-free text as illustrated in Table 3.

**Table 3:** Percentage distribution of the researchers according to their opinions toward pros of using ChatGPT (*n* = 1001)

S.	Researchers’ opinion of using ChatGPT	No		Maybe		Yes	
		No.	%	No.	%	No.	%
1	ChatGPT helps to overcome language barriers.	74	7.4	208	20.8	719	71.8
2	It can enhance efficiency in scientific writing and translation purposes.	90	9.0	203	20.3	708	70.7
3	ChatGPT can help to summarize research papers.	85	8.5	185	18.5	731	73.0
4	It can facilitate the work of researchers and help in data collection	61	6.1	162	16.2	778	77.7
5	ChatGPT helps to save time.	53	5.3	130	13.0	818	81.7
6	It can help to provide easily accessible and understandable information	62	6.2	170	17.0	769	76.8
7	ChatGPT can help to increase efficiency and reduce errors.	92	9.2	215	21.5	694	69.3
8	It considered cost saving.	108	10.8	194	19.4	699	69.8
9	ChatGPT can help in academic writing.	95	9.5	208	20.8	698	69.7
10	It characterized by well-organized content with decent references.	111	11.1	220	22.0	670	66.9
11	It promotes a motivation to write.	125	12.5	192	19.2	684	68.3
12	It has original, precise, and accurate responses with systematic approach.	94	9.4	224	22.4	683	68.2
13	ChatGPT can be a useful tool for researchers.	64	6.4	161	16.1	776	77.5
14	It is useful for literature review, can help in data analysis, and in translation.	70	7.0	190	19.0	741	74.0
15	It has more productivity among researchers.	82	8.2	190	19.0	729	72.8
16	It has the ability to generate plagiarism-free text.	142	14.2	259	25.9	600	59.9
17	It can improve health literacy with better patient outcomes.	93	9.3	208	20.8	700	69.9

Table 4. illustrated that 56.4% of nursing researchers believed that ChatGPT had ethical concerns (ghostwriting), 49.5% doubted of its accuracy, 50.9% thought that it can cause citation problems, 45.1% supposed that it can affect research quality, and 49.7% reported that ChatGPT may lead to decreased critical thinking and creativity.

**Table 4:** Percentage distribution of the researchers according to their opinions toward cons of using ChatGPT (*n* = 1001)

Q	Researchers’ opinion of using ChatGPT	No		Maybe		Yes	
		No.	%	No.	%	No.	%
1.	It had ethical concerns (ghostwriting)	178	17.8	258	25.8	565	56.4
2.	Its’ accuracy is doubtful	233	23.3	273	27.3	495	49.5
3.	It can cause citation problems	203	20.3	288	28.8	510	50.9
4.	Its’ content is not original	473	47.3	249	24.9	279	27.9
5.	It has incorrect answers that sound plausible	407	40.7	266	26.6	328	32.8
6.	It is associated with risk of plagiarism	347	34.7	255	25.5	399	39.9
7.	It has risk of bias	363	36.3	267	26.7	371	37.1
8.	Several ChatGPT responses lacked depth and insight	299	29.9	260	26.0	442	44.2
9.	It has copyright infringements possibility	342	34.2	259	25.9	400	40.0
10.	It is associated with data insecurity	379	37.9	236	23.6	386	38.6
11.	ChatGPT has lack of personal experience highlights	349	34.9	229	22.9	423	42.3
12.	Its’ Knowledge restricted to the period before 2021	377	37.7	265	26.5	359	35.9
13.	It has concerns about misuse in the academia (Academic dishonesty)	319	31.9	244	24.4	438	43.8
14.	It is characterized by compromised research quality	298	29.8	252	25.2	451	45.1
15.	ChatGPT may lead to decreased critical thinking and creativity in science	281	28.1	223	22.3	497	49.7
16.	It lacks a human researcher’s depth of knowledge and expertise.	289	28.9	233	23.3	479	47.9

The overall opinion of nursing researchers illustrated that 81.4% exhibited positive view concerning pros of using ChatGPT. While, 44.7% were announced for cons as revealed in Table 5.

**Table 5:** Descriptive analysis of the studied participants according to overall scores for researchers’ opinion of using ChatGPT (*n* = 1001)

Researchers’ opinion of using ChatGPT	Score Range	Total Score	Average score	Percent Score
		Mean ± SD.	Mean ± SD.	Mean ± SD.
Pros	(0–34)	27.7 ± 6.7	1.6 ± 0.4	81.4 ± 19.7
Cons	(0–32)	14.3 ± 8.4	0.9 ± 0.5	44.7 ± 26.3

SD: **Standard deviation**

Table 6. shows the barriers of applying of AI in health care researches. It reveals that 68.8% of nursing researchers announced that it may cause harmful or wrong decisions, 68.6% stated that it may result in medicolegal implications, 67.8% resisted to adopt AI Chat bot in health care decisions, and 65.6% worried about patients’ confidentiality.

**Table 6:** Percentage distribution of the participants according to barriers of applying artificial intelligence in health care researches (*n* = 1001)

S.	Barriers of applying artificial intelligence in health care researches	No.	%
1	AI chat bots are not yet well-developed.	589	58.8
2	It is not available for me.	546	54.5
3	It lacks of credibility / Unknown source of information that feeds that data to the AI model.	611	61.0
4	I do not know which AI model can be used in healthcare researches.	647	64.6
5	I worry about my patients confidentiality when using AI in their care.	657	65.6
6	I am worried AI will take over the human roles in healthcare researches.	646	64.5
7	I am worried it may recommend harmful or wrong decisions.	689	68.8
8	I’m not familiar with using AI-Chat bots.	600	59.9
9	Resistance of some healthcare providers to adopt AI Chat bot in health care decisions.	679	67.8
10	Medicolegal implications of using AI for patients care presented.	687	68.6

Table 7. portrays a statistically significant relation between age (18 < 30 years) and researchers’ opinion about pros of using ChatGPT with F (p) = 15.624^*^ (< 0.001^*^). Also, a statistically significant relation between female gender and researchers views of cons of using ChatGPT with t (p) = 3.065^*^ (0.002^*^). Moreover, doctorate degree researchers illustrated high percent relation with pros of ChatGPT but not reach statistically significant relation with mean = 83.7. Besides, researchers who had good computer knowledge exhibits high percent relation with pros of using ChatGPT with mean = 82.5.

**Table 7:** Relation between percent score for overall researchers’ opinion of using ChatGPT and demographic and technical data (*n* = 1001)

Demographic data	No.	Percent score for overall researchers’ opinion of using ChatGPT	
		Pros	Cons
		Mean ± SD.	Mean ± SD.
**Age**			
18 < 30 years	866	82.7 ± 19.1	45.2 ± 26.9
30 < 40 years	120	72.2 ± 21.7	42.9 ± 21.7
40 < 60 years	15	79.6 ± 16.9	33.3 ± 21.7
F (p)		15.624^*^ (< 0.001^*^)	1.845 (0.159)
Effect size (η^2^)		0.030	**–**
**Gender**			
Male	360	82.8 ± 19.9	41.3 ± 27.8
Female	641	80.6 ± 19.6	46.7 ± 25.2
t (p)		1.685 (0.092)	3.065^*^ (0.002^*^)
Effect size (Cohen’s d)		–	0.205
**Level of education**			
Bachelor’s Degree	561	81.3 ± 18.4	43.8 ± 24.9
Master	275	81.0 ± 21.5	46.8 ± 26.7
Doctorate	122	83.7 ± 19.8	46.6 ± 30.4
Ass. Prof.	21	76.2 ± 28.3	39.0 ± 28.6
Professors	22	82.6 ± 19.8	39.6 ± 27.8
F (p)		0.822 (0.511)	1.229 (0.297)
**Specialty**			
Medical Surgical nursing	344	81.7 ± 20.1	45.7 ± 26.3
Critical care nursing	204	80.4 ± 18.3	41.3 ± 22.7
Obstetric nursing	55	81.1 ± 17.9	46.9 ± 26.4
Pediatric nursing	106	82.1 ± 20.2	44.0 ± 30.1
Community health nursing	133	83.2 ± 19.7	47.0 ± 28.3
Administration nursing	108	80.3 ± 20.3	44.8 ± 24.5
Psychiatric nursing	51	80.3 ± 22.9	45.1 ± 28.6
F (p)		0.404 (0.877)	0.884 (0.506)
**Level of computer knowledge and expertise**			
Good	632	82.5 ± 19.7	45.0 ± 27.2
Fair	247	80.0 ± 20.0	45.9 ± 24.9
Poor	122	79.0 ± 18.7	40.9 ± 23.9
F (p)		2.505 (0.082)	1.546 (0.214)

SD: **Standard deviation t: Student t-test F**: **F for One-way ANOVA test**

p: p value for comparison between the studied categories Ass. Prof. Associate professor

*: Statistically significant at *p* ≤ 0.05

## Discussion

As we are in the AI era, authors found it is crucial to ask the participants about their opinion. The current study identified nursing researchers’ view of using ChatGPT. To give a snapshot of the demographic findings, statistical analysis showed that more than two thirds were between 18 to less than 30 years old and two thirds of them were female. The young age of the participants reflects young generations’ interest in technology that illustrated in the study findings as two thirds of the participants had good knowledge and expertise of computer. The present findings supported by a study [17] about the impact of ChatGPT on knowledge sharing among language research students and reported that more than two thirds of nursing researchers were between 18 to less than 30 years old, also half of them were female.

Concerning the current research and using ChatGPT, more than two thirds of the participants working on current research less than one year and more than half of them had experience with ChatGPT and used it in their current research. That may be related to technology revolution and the ease access of the AI tools such as ChatGPT and variety of resources available on it to decrease waste of time and effort for researchers. Similarly, to the findings of a study [18] on knowledge, perceptions and attitude of researchers towards using ChatGPT in research and revealed that more than two thirds of the researchers have good knowledge about ChatGPT. While, the same study [18] revealed inconsistency with the current research that only more than 10% of the researchers used ChatGPT in their research.

As regards the participants’ opinions toward pros of using ChatGPT, the present study showed that the vast majority of nursing researchers exhibits positive view toward pros of using ChatGPT. The majority of them reported that ChatGPT helps to save time, more than two thirds stated that ChatGPT can be a useful tool for researchers, and helps to provide easily accessible and understandable information. While more than half stated that ChatGPT able to generate plagiarism-free text, that stimulates the researchers to the utilize ChatGPT.

Moreover, the findings of this study strengthen the argument that AI-based technologies can be used as effective learning tools to broaden researchers’ horizons and improve their analytical abilities. These positive results also pave the way for a wider incorporation of technology into technology-based education in consistent with a study [19] about “pros and cons of using ChatGPT in academic writing instruction”, and the study revealed that researchers exhibit positive opinions toward the pros of using ChatGPT as it helps to provide easily accessible and understandable information. Also, a study [20] about “prospectives on the use of ChatGPT in education: pros and cons with a classical approach,” agreed with the current results that ChatGPT saved time and it is a useful tool for researchers.

In spite of the participants’ positive view toward ChatGPT, they thought that it may be accompanied with drawbacks as the current study illustrated that more than half of nursing researchers believed that ChatGPT had ethical concerns (ghostwriting). Besides, less than half of them doubted its accuracy, half of them thought that it could cause citation problems, one third of them supposed that it could affect research quality, and almost half reported that ChatGPT may lead to declined critical thinking and inventiveness which may be one of the major challenges that scientific writers face. They must communicate technical and complicated material in a way that a larger audience may comprehend. Considering the volume of technical terms, jargon, and specialized language used in scientific writing, this is especially crucial.

One of the major limitations of ChatGPT in scientific writing is that it lacks a human researcher’s depth of knowledge and expertise. It can help humans who are the experts [21]. Scientific writing necessitates a deep comprehension of intricate concepts, theories, and experiments carried out in the field of study. ChatGPT, in contrast, utilizes algorithms and existing data to formulate replies, which may be insufficient when it comes to formulate scientific papers. Therefore, a good scientific writer should be able to strike a balance between the need for technical accuracy and the need for effective communication.

Artificial intelligence can assist during the editing and proofreading phases of scientific writing, but it cannot fully replace human proficiency. Furthermore, ChatGPT is unable to determine which information is properly related compared to what is not related. Moreover, a study [22] corroborated the negative aspects of using ChatGPT in scientific writing, where precision is paramount, and even a small mistake can have serious consequences in the research domain. Although scientific writing demands exactness and carefulness, ChatGPT often has difficulty in balancing the needs for relevance and precision. It may produce information that lacks importance, which could also negatively affect research outcomes. Finally, effective scientific writing demands that authors follow particular formatting and referencing standards. The structured scientific work is crucial for presenting research in an orderly and thorough way. The difficulty ChatGPT experiences in understanding these intricate formatting and referencing requirements can hinder its ability to produce research articles that comply with scientific writing standards.

Even ChatGPT has advantages, ethical considerations, and the need for human oversight cannot be ignored. Researchers should exercise caution when utilizing ChatGPT, ensuring that the outputs are validated and cross-verified with reputable sources. Future research should focus on addressing these concerns, developing robust validation methods. These findings agree with a study [23] about pros and cons of using ChatGPT in medical research and publishing – a comprehensive review, also a study [24] about “potential benefits and challenges of ChatGPT in future nursing education,” both reported that majority of nursing researchers have a positive view about pros of using ChatGPT. While a minority of them focus on cons of using ChatGPT.

Although the pros of AI as illustrated in participants’ opinion for ChatGPT utilization, there are some barriers for applying of AI in health care research. Almost two thirds of nursing researchers announced that it may cause harmful or wrong decisions, may result in medicolegal implications, and resisted to adopt AI Chat bot in health care decisions. Furthermore, two thirds of them worried about patients’ confidentiality. Another study [25] titled " Analysing the impact of artificial intelligence in ESL Education: a systematic review” agreed with the current study and stated that the use of ChatGPT in academic research is becoming more common among students. This technology has helped students cope with time constraints in completing research tasks, but it also raises concerns about academic ethics, such as plagiarism and research misconduct. Additionally, in a study [26] about using ChatGPT in nursing: scoping review of current opinions that researchers, institutions, and administrations still need to critically examine its accuracy, safety, and privacy, as well as academic misconduct and potential ethical issues that it may cause before applying ChatGPT to practice.

The current study indicated that there was a statistically significant relation between younger age and researchers’ opinion about pros of using ChatGPT. This may be due to increased younger researchers’ interests concerning technology as the study illustrated that those who had good computer knowledge exhibits high percent relation with pros of using ChatGPT. Besides, a statistically significant relation between female gender and researchers views of cons of using ChatGPT. Moreover, doctorate degree researchers illustrated high percent relation with pros of ChatGPT but not reach statistically significant relation. The literature about the pros and cons of ChatGPT confirmed the results from the recent research [27, 17, 28, 29]. All of those studies reported that AI technology has both sides, a positive side and a dark side. It depends on age, gender, education, and the ethics of the researcher who is responsible for using it.

## Conclusion

ChatGPT can revolutionized academic writing, but can’t replace researchers. The current study findings conclude that 81.4% of participants had a positive view for the pros of ChatGPT utilization. While, 44.7% were announced for the cons. Furthermore, 68.8% of nursing researchers announced that it may cause wrong decisions, 68.6% stated that it may result in medicolegal implications, 65.6% worried about patients’ confidentiality, and 67.8% resisted to adopt AI Chat bot in health care decisions.

### Recommendations

As AI tools have both aspects “the bright aspect and dark aspect” we should use it cautiously to achieve the most benefit.

1. Evaluate AI’s performance continuously and incorporate feedback from nursing students, educators, and professionals to remain up-to-date with the latest nursing research, best practice, and technological advances.
2. Strengthen its capacity to support hands-on skills training by developing features that facilitate clinical simulations, real-time feedback, and remote supervision. This will help bridge the gap between theoretical knowledge and practical skills in nursing research.
3. Incorporate features that present complex patient scenarios, promote evidence-based decision making and encourage reflective practice to support critical thinking and decision making.
4. Be aware not to use ChatGPT in clinical nursing care.
5. Training for ethics to avoid ghostwriting.

### Limitations of the study

Many of the participants were younger, so the authors were forced to present their opinions, whereas if the proportion of older researchers had been larger, the results might have changed.

The study is single-country scope.

## Electronic supplementary material

Below is the link to the electronic supplementary material.

Supplementary Material 1

## Data Availability

The datasets generated and analyzed during the current study are not publicly available because confidentiality pledged by the authors for participants while obtaining approval to conduct the research but are available from the corresponding author on reasonable request.

## Abbreviations

ChatGPT: Chat Generative Pre-Trained Transformers
AI: Artificial intelligence
NLP: Natural language processing
REC: Research ethics committee
