# Supplementary material for: Chat generative pre-trained transformers era: pros and cons between nursing researchers in Egypt
Source: BMC Nurs. 2025 Jun 24;24:667. doi: 10.1186/s12912-025-03332-1 (PMC12186332; doi:10.1186/s12912-025-03332-1)
Supplement: Supplementary file 1 — Supplementary Material 1 [file 12912_2025_3332_MOESM1_ESM.pdf]

Chat GPT

Your contribution is greatly valued

* Required

1. **Dear colleagues,**

   You are invited to participate in this survey, even if you did not use Chat GPT before.
   It will take approximately 2-3 minutes of your time. Your participation is completely voluntary.
   The responses are completely anonymous and confidential, and researchers will not be able to identify participants. We will use the collected information for research purposes only.
   Are you agree to participate *

   - ◯ Yes
   - ◯ No

2. **Age e.g. (25):** *

   ____

3. **Gender** *

   - ◯ Male
   - ◯ Female

4. **Level of education:** *

   - ◯ Bachelor's Degree
   - ◯ Master
   - ◯ Doctorate
   - ◯ Assist. prof.
   - ◯ Prof.

5. **University:** *

- ◯ Ain Shams University
- ◯ Al-Azhar University
- ◯ Alexandria University
- ◯ Assiut University
- ◯ Aswan University
- ◯ Banha University
- ◯ Beni-Suef University
- ◯ Cairo University
- ◯ Damanhour University
- ◯ Damietta University
- ◯ Egypt-Japan University of Science and Technology
- ◯ Fayoum University
- ◯ Helwan University
- ◯ Kafrelsheikh University
- ◯ Luxor University
- ◯ Mansoura University
- ◯ Military Technical College
- ◯ Minia University
- ◯ Minufiya University
- ◯ New Valley University
- ◯ Port Said University
- ◯ Sadat Academy for Management Sciences
- ◯ Sohag University
- ◯ South Valley University
- ◯ Suez Canal University
- ◯ Suez University
- ◯ Tanta University
- ◯ University of Sadat City
- ◯ Zagazig University
- ◯ Other

6. If other, enumerate.....

7. **Speciality:** *

- Medical Surgical nursing
- Critical care nursing
- Obstetric nursing
- Pediatric nursing
- Community care nursing
- Administration nursing
- Psychiatric nursing

8. **How would you rate your level of computer knowledge and expertise?** *

- Good
- Fair
- Poor

9. **Do you have any experience with ChatGPT?** *

- Yes
- No

10. **What do you believe ChatGPT to be?** *

- Online expert panel discussion
- A type of artificial intelligence
- A predesigned answers of commonly asked questions
- I am not sure

11. **How long have you been working on current research?** *

- ◯ Less than 1 years
- ◯ 1<2 years
- ◯ 2<5 years
- ◯ 5<10 years
- ◯ more than 10 years

12. **Did you use ChatGPT in current research?** *

- ◯ Yes
- ◯ No

13. **Researchers' opinion of using chat GPT:**
Please, read the phrases carefully and select your opinion
*

	Yes	No	Maybe
1. ChatGPT helps to overcome language barriers	○	○	○
2. Improved efficiency in scientific writing; translation purposes	○	○	○
3. ChatGPT can help to summarize research papers	○	○	○
4. It can facilitate the work of researchers; it can help in data collection	○	○	○
5. ChatGPT helps to save time	○	○	○
6. It can help to provide easily accessible and understandable information	○	○	○
7. ChatGPT can help to increase efficiency; it helps to reduce errors	○	○	○
8. It considered cost saving	○	○	○
9. ChatGPT can help in academic writing	○	○	○
10. Well-organized content with decent references; a free package	○	○	○
11. Promoting motivation to write	○	○	○
12. Original, precise, and accurate responses with systematic approach	○	○	○
13. ChatGPT can be a useful tool for researchers	○	○	○

	Yes	No	Maybe
14. Useful for literature review; can help in data analysis; can help in translation	○	○	○
15. More productivity among researchers	○	○	○
16. Ability to generate plagiarism-free text	○	○	○
17. Improved health literacy with better patient outcome	○	○	○
18. Ethical concerns (ghostwriting)	○	○	○
19. Doubtful accuracy	○	○	○
20. It can cause citation problems	○	○	○

14. **Researchers' opinion of using chat GPT:**
Please, read the phrases carefully and select your opinion *

	yes	No	Maybe
21. Content is not original	○	○	○
22. Incorrect answers that sound plausible	○	○	○
23. Risk of plagiarism	○	○	○
24. Risk of bias	○	○	○
25. Several chat GPT responses lacked depth and insight	○	○	○
26. Copyright infringements possibility	○	○	○
27. It is associated with data insecurity	○	○	○
28. Lack of personal experience highlights	○	○	○
29. Restricted knowledge to the period before 2021	○	○	○
30. Concerns about misuse in the academia (Academic dishonesty)	○	○	○
31. Compromised research quality	○	○	○
32. ChatGPT may lead to decreased critical thinking and creativity in science	○	○	○
33. lacks a human researcher's depth of knowledge and expertise.	○	○	○

15. **Barriers of applying artificial intelligence in health care researches**
What do you believe to be the key barriers of applying AI (Artificial Intelligence) in healthcare researches at this time? *

	Yes	No
AI chatbots are not yet well-developed	◯	◯
Not available for me	◯	◯
Lack of credibility / Unknown source of information that feeds that data to the AI model	◯	◯
I do not know which AI model can be used in healthcare researches	◯	◯
I worry about my patients confidentiality if I used AI in their care	◯	◯
I am worried AI will take over the humans roles in healthcare researches	◯	◯
I am worried it may recommend harmful or wrong decisions	◯	◯
Not familiar with using AI-Chatbots	◯	◯
Resistance of some healthcare providers to adopt AI Chatbot in health care decisions	◯	◯
Medicolegal implications of using AI for patients care	◯	◯

16. **If other (please specify)**

17. **Overall opinion about ChatGPT**
How at ease would you feel using Chat GPT in your nursing research?
*

◯ Very comfortable

◯ Somewhat comfortable

◯ Not comfortable at all

18. **What potential effects do you see ChatGPT to have on the future of healthcare researches?** *

◯ Improve the future of healthcare

◯ Have no impact

◯ Worsen the future of healthcare

19. **Considering that you have not yet used ChatGPT in your healthcare practice: Do you intend to use in the future?** *

◯ Yes

◯ No

This content is neither created nor endorsed by Microsoft. The data you submit will be sent to the form owner.

Microsoft Forms
